# Canine hookworms in the Philippines—Very common but very much neglected in veterinary research

**DOI:** 10.3389/fvets.2023.1297962

**Published:** 2023-11-29

**Authors:** Jan Clyden B. Tenorio

**Affiliations:** ^1^Department of Veterinary Paraclinical Sciences, College of Veterinary Medicine, University of Southern Mindanao, Cotabato City, Philippines; ^2^Department of Tropical Medicine, Faculty of Medicine, Khon Kaen University, Khon Kaen, Thailand

**Keywords:** *Ancylostoma*, One Health, parasitology, soil-transmitted helminths, neglected tropical diseases

Hookworm infections caused by *Ancylostoma caninum, A. ceylanicum, A. braziliense*, and *Uncinaria stenocephala* continue to be a significant threat to canine health globally (1, 2). In Asia, it is estimated that hookworm infections have a prevalence of around 35% among different species of canids endemic to the continent, with approximately 41% of domesticated dogs infected (3). Moreover, in Southeast Asia, recent reports have proven that the most common dog hookworm is the zoonotic *A. ceylanicum*, with *A. caninum* being a close second (4, 5). Conversely, *A. ceylanicum* is now regarded as the region's second most prevalent human hookworm, next only to *Necator americanus* (4, 6). Canine hookworms are of One Health significance since they have been noted to cause deleterious infection consequences in humans: *A. caninum* causes eosinophilic enteritis, *A. braziliense* is associated with “creeping eruptions,” and *A. ceylanicum* infections is a zoonosis that can circulate between humans and companion animals (1, 7, 8). This presents a One Health scenario where zoonotic hookworms from dogs cause a significant public health concern among neglected, impoverished communities (9).

In the Philippines, historical accounts have reported that *A. caninum, A. ceylanicum*, and *A. braziliense* have infected dogs and inhabitants in the country (10–12). Among these, only *A. ceylanicum* has been molecularly confirmed to infect both dogs and humans from the same community (13). Unfortunately, there are only five articles that have been published in the peer-reviewed and online gray literature from 2000 to 2023 regarding the epidemiology of canine hookworms in the Philippines. The reported prevalence ranges from 8% in urban areas such as Manila and Laguna Province to 48% in rural endemic areas of Mindanao (14, 15). For a country that has an estimated dog population of 23 2,900, 000 (16), roughly 11 million dogs would potentially be affected by hookworm infections. Figure 1 summarizes the results and geographic distribution of these studies. Aside from these recent reports, we know nothing about the epidemiology of canine hookworm infections.

**Figure 1:**
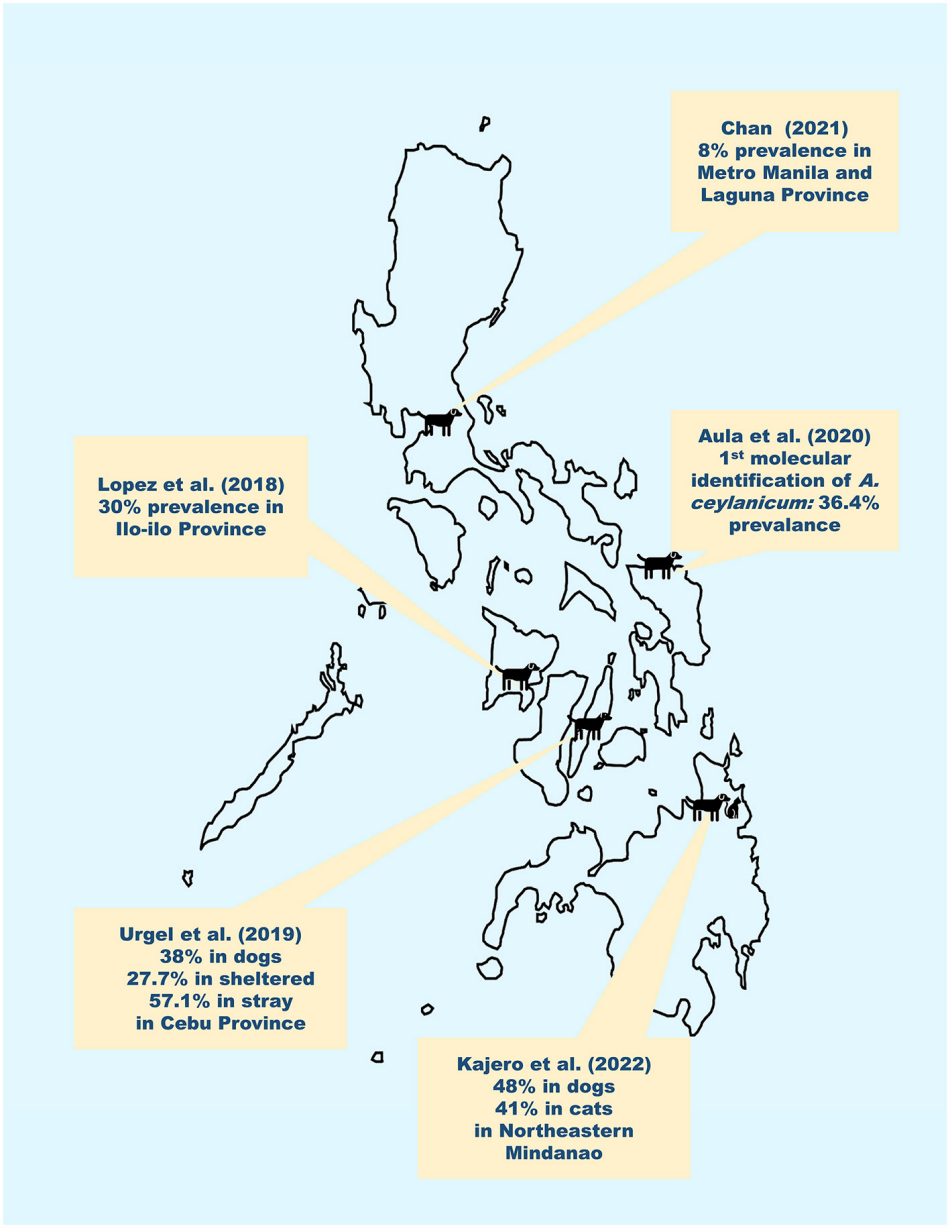
Results and geographical distribution of recent published reports (2000–2023) of canine hookworm infections in the Philippines. Recent reports by Chan (15), Lopez et al. (17), Urgel et al. (18), Aula et al. (13) and Kajero et al. (14) indicate that canine hookworm infections are between 8% to 48% in the Philippines.

I posit several possible reasons as to why there is a paucity of peer-reviewed literature regarding canine hookworm infections in the Philippines, which include the following:

1. Hookworm infections are neglected because these are not seen as a pressing canine health concern compared to other prevalent infectious diseases (e.g., canine ehrlichiosis and canine parvoviral enteritis).
2. The clinical management of canine gastrointestinal parasites is relatively straightforward due to broad-spectrum, combination anthelmintics. Resistance to any anthelmintic among canine hookworms has yet to be reported in the country, at least in the peer-reviewed literature. However, multidrug-resistant hookworms have already been noted in the United States, Australia, Brazil, New Zealand, and Canada (19–23).
3. Most cases of infections do not present overt clinical signs; hence, most owners are not aware that their pets are infected, which adds to the general neglect of canine hookworms. However, it must be noted that severe acute infections among puppies cause significant pathology (e.g., anemia and bloody diarrhea) and even death (24).
4. The unequal access to veterinary care among financially challenged dog owners results in poor knowledge of the damage caused by canine hookworms and the neglect of infections. This is of particular concern since hookworm infections are most likely to occur in resource-lacking endemic areas (25).
5. The notion that canine hookworm infections are ubiquitous undermines the value of researching their occurrence, risk factors, and control. This notion of undervaluing results in numerous gaps in research (e.g., drug resistance, infection epidemiology, and transmission dynamics) that continue to be unexplored in the country.
6. Student theses that investigate this problem are often never published in peer-reviewed journals; most Filipino universities do not have online repositories that store and make these research results available.

The dearth of research on canine hookworms in the Philippines presents some important research gaps and opportunities, which include the following:

1. There is a need to molecularly confirm the species of hookworm that currently affect dogs in the country, so we know which hookworm species occur in which area.
2. The epidemiology and risk factors of canine ancylostomiasis in most areas of the Philippines remain poorly understood. Epidemiological data will enable clinical practitioners to develop health programs that consider factors affecting hookworm infections, which will be effective for their canine patients.
3. The zoonotic nature of *A. ceylanicum* presents a One Health risk among Filipinos and companion animals that should be studied. The transmission dynamics and occurrence of this important hookworm are still poorly understood in the country. Research results addressing this issue can be used to optimize the Integrated Helminth Control Program of the Philippine Department of Health.
4. Research on soil contamination of canine hookworms and other geohelminths (i.e., *Toxocara* and *Trichuris*) is lacking in many endemic areas in the country. This research gap may be of importance since high *Toxocara* seroprevalence occurs among children exposed to areas with contaminated soils, which have been previously reported in the Philippines (26, 27).
5. The efficacy of treatment and control plans should be re-evaluated as unforeseen resistance to commonly used anthelmintics may have already occurred. Anthelmintic drug efficacy determination can be done based on the reduction of fecal egg count reduction test (FECRT) using parasitological assays (e.g., McMaster or Mini FLOTAC techniques) or molecular techniques (e.g., quantitative PCR) (28, 29). Moreover, specific gene mutations involved in resistance can be assessed, such as single nucleotide polymorphisms in the β*-tubulin isotype 1* gene for benzimidazole resistance. SNPs reported among populations of *A. caninum* include those that occur at positions 134 (CAA/glutamine → CAT/histidine), 167 (TTC, TTT/phenylalanine → TAC, TAT/tyrosine), 198 (GAG, GAA/glutamic acid → GCG, GCA/alanine), and 200 (TTC/ phenylalanine → TAC/tyrosine or TTC/ phenylalanine → TTA/leucine) (23, 30–32).
6. Owner awareness of the risks and harms of canine hookworms should be assessed and subsequently improved. Barriers to accessing essential veterinary care for dog owners should also be investigated.

To end, research on the epidemiological features of canine hookworms on the national scale remains wanting in the Philippines. The impact of this important parasitosis is also poorly understood, leading to neglect of its potential One Health consequences. Therefore, it is the intent of this piece to present the dearth of veterinary research in canine hookworms in the country and the opportunities it presents in the hopes that actions can be sparked among the Filipino veterinary community.

## Author contributions

JT: Conceptualization, Investigation, Writing – original draft, Visualization.
